# BMI1 nuclear location is critical for RAD51-dependent response to replication stress and drives chemoresistance in breast cancer stem cells

**DOI:** 10.1038/s41419-022-04538-w

**Published:** 2022-02-02

**Authors:** Violette Azzoni, Julien Wicinski, Manon Macario, Martin Castagné, Pascal Finetti, Katerina Ambrosova, Célia D. Rouault, Arnaud Sergé, Anne Farina, Emilie Agavnian, Sergiu Coslet, Emmanuelle Josselin, Arnaud Guille, José Adelaide, Emmanouil Zacharioudakis, Rémy Castellano, Francois Bertucci, Daniel Birnbaum, Raphael Rodriguez, Emmanuelle Charafe-Jauffret, Christophe Ginestier

**Affiliations:** 1 Aix-Marseille Univ, Inserm, CNRS, Institut Paoli-Calmettes, CRCM, Epithelial Stem Cells and Cancer Lab, “Equipe labellisée Ligue Contre le Cancer”, Marseille, France; 2 Aix-Marseille Univ, Inserm, CNRS, Institut Paoli-Calmettes, CRCM, Predictive Oncology, “Equipe labellisée Ligue Contre le Cancer”, Marseille, France; 3 Aix-Marseille Univ, Inserm, CNRS, Institut Paoli-Calmettes, CRCM, leuko/stromal interactions in normal and pathological hematopoiesis Lab, Marseille, France; 4 Aix-Marseille Univ, Inserm, CNRS, Institut Paoli-Calmettes, CRCM, Experimental Pathology Platform, Marseille, France; 5 Aix-Marseille Univ, Inserm, CNRS, Institut Paoli-Calmettes, CRCM, TrGET Plateform, Marseille, France; 6 Institut Curie, CNRS, INSERM, PSL Research University, Chemical Cell Biology Group, Paris, France

**Keywords:** Breast cancer, Cancer stem cells

## Abstract

Replication stress (RS) has a pivotal role in tumor initiation, progression, or therapeutic resistance. In this study, we depicted the mechanism of breast cancer stem cells’ (bCSCs) response to RS and its clinical implication. We demonstrated that bCSCs present a limited level of RS compared with non-bCSCs in patient samples. We described for the first time that the spatial nuclear location of BMI1 protein triggers RS response in breast cancers. Hence, in bCSCs, BMI1 is rapidly located to stalled replication forks to recruit RAD51 and activate homologous-recombination machinery, whereas in non-bCSCs BMI1 is trapped on demethylated 1q12 megasatellites precluding effective RS response. We further demonstrated that BMI1/RAD51 axis activation is necessary to prevent cisplatin-induced DNA damage and that treatment of patient-derived xenografts with a RAD51 inhibitor sensitizes tumor-initiating cells to cisplatin. The comprehensive view of replicative-stress response in bCSC has profound implications for understanding and improving therapeutic resistance.

## Introduction

Cancer cells are characterized by a loss of control mechanisms for DNA replication, which causes cellular stress named replication stress (RS). It results in the generation of inefficient DNA replication, leading to genome instability and ultimately genomic alterations [1]. This phenomenon, referred as a hallmark of cancer, is a central driver to tumor initiation and progression [2]. RS fuels an evolutionary process at the cell level, driven by genetic and epigenetic alterations that lead to tumor-cell heterogeneity [3]. It was first estimated that genomic alterations accumulating during tumor progression followed the rules of a Darwinian selection. However, accumulating evidences suggest that an additional layer of functional diversity collaborates with RS to shape tumor heterogeneity [4]. Hence, each genomic clone within a tumor represents a complex ecosystem where a subpopulation of tumorigenic cells, the so-called cancer stem cells (CSCs), appears to orchestrate the evolutionary selection in cancer [4, 5]. A direct consequence is the pivotal role of CSCs in leading clinical evolution. We and others have clearly established a positive association between CSC burden and disease progression or therapeutic failure [6–9]. Thus, it is of major interest to gain insight into the collaboration between stemness and RS in order to better understand the evolution of cancers’ clonal architecture during tumor progression and propose new approaches to overcome therapeutic failures.

Several studies revealed an increased expression of many DNA-repair genes in CSCs from different tissues compared with bulk tumor cells [10–13]. This key cellular function might be inherited from normal adult stem cell programs that limit RS through the activation of DNA-damage response (DDR) to avoid organ failure, premature aging, or cancer formation [14]. Moreover, genes involved in genome-stability maintenance seem to be preponderant in the control of cellular differentiation, fine-tuning tissue homeostasis [15, 16]. In tumors, such a robust DDR in CSCs acts as a modulator of survival by limiting a high level of RS in cells with profoundly altered genomes. Hence, cumulative dysregulation of DNA-repair programs with uncontrolled cell-cycle checkpoints can aggravate the toxicity of replicative lesions and jeopardize cancer-cell survival [15, 17]. A consequence of prolonged RS response in CSCs may be the acquisition of resistance to DNA-damage-inducing agents. Indeed, CSCs appear to survive conventional cancer therapies such as radiotherapy or chemotherapies [10, 18, 19]. In this context, understanding the underlying mechanisms that control RS response in CSCs may offer a unique opportunity to sensitize tumorigenic cells to therapies.

In this study, we interrogated RS through the prism of tumor-cell heterogeneity by measuring its level in breast CSCs (bCSCs) compared with mature cancer cells and revealed that bCSCs displayed a limited level of RS due to a constitutive activation of BMI1/RAD51 axis. We show that BMI1/RAD51 axis play a pivotal role in the therapeutic resistance of bCSCs to DNA-damaging agents and provided evidence that inhibiting RAD51 can chemosensitize bCSCs.

## Results

### Breast CSCs present a limited level of replication stress (RS)

**Fig. 1:**
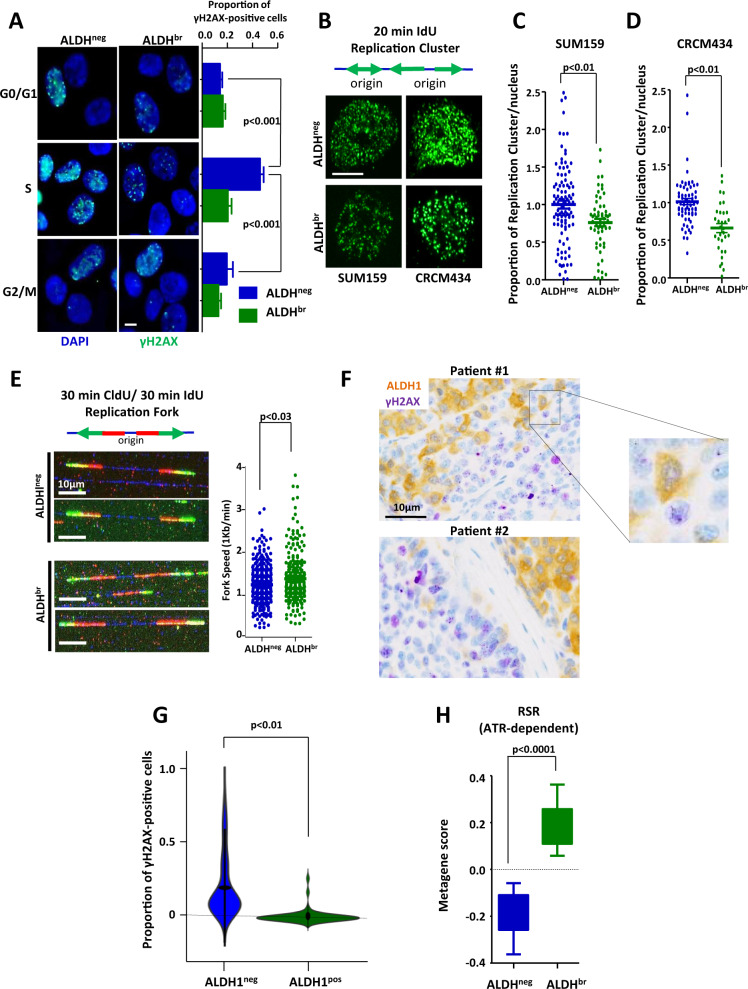
ALDH^br^ breast CSCs presented reduced replication stress in vitro and in patient samples. **A** Representative images of γH2AX staining (green foci) in ALDH^br^ and ALDH^neg^ cells sorted according to their cell-cycle phase (G0/G1, S, and G2/M). Nuclei are counterstained with DAPI (blue staining). Scale bar: 5 μm. Bar plots represent the proportion of γH2AX-positive cells for each cell subpopulation. **B** SUM159 and CRCM434 cells were pulse-labeled with iododeoxyuridine (IdU) for 20 min and fixed. Active replication clusters were detected by immunostaining for IdU (green foci). Scale bar: 5 μm. **C**, **D** Bee-swarm plots representing the proportion of replication clusters per nucleus in each cell subpopulation (ALDH^br^ and ALDH^neg^) isolated from SUM159 (**C**) and CRCM434 (**D**). **E** Cells were sequentially labeled with chlorodeoxyuridine (CldU) and IdU for 30 min each. The replication forks progress in both directions at the same rate and integrate the analogs. Representative images of combed DNA molecules are shown. CldU (green staining) and IdU (red staining) were detected using specific antibodies and DNA is counterstained with DAPI (blue staining). Scale bar: 10 μm. Bee-swarm plots represent the distribution of fork speed in ALDH^br^ and ALDH^neg^ cells. **F** Representative tumor-sample sections with γH2AX (purple staining) and ALDH (brown staining) immunostaining. **G** Violin plot representing the proportion of γH2AX-positive cells in ALDH1^pos^ and ALDH1^neg^ cell subpopulations detected on breast tumor samples. Scale bar: 10 μm. **H** Box plot representing the gene expression level of the replication-stress-response metagene in ALDH^br^ and ALDH^neg^ cells isolated from PDXs. Statistical test used is Student’s *t*-test. Data represent mean ± SD.

To evaluate the RS level in breast CSCs (bCSCs) compare with non-bCSCs, we sorted SUM159 cells based on their ALDH enzymatic activity [6, 11] and cell cycle stages (Supplementary Fig. 1). Sorted cells were stained for the detection of the phosphorylated histone variant H2AX (γH2AX) testifying DNA damages. Non-bCSCs (aka ALDH^neg^) presented a significant increase of γH2AX-positive cells in S phase compare with other cell-cycle phases, suggesting DNA-damage accumulation at stalled forks (Fig. 1A). Conversely, bCSCs (aka ALDH^br^) showed a weak and steady proportion of γH2AX-positive cells through all cell-cycle stages, including S phase, vouching for a limited RS in bCSCs. We next counted the number of active replication clusters in sorted cells pulse-labeled with the thymidine analog iododeoxyuridine (IdU) (Fig. 1B). We quantified the number of IdU foci in each nucleus using an automated image-analysis tool [20]. bCSC displayed lower numbers of replication foci compared with non-bCSC (Fig. 1C). Similar results were also observed in tumor cells isolated from a PDX model (CRCM434) established directly from primary breast tumor [9] (Fig. 1D). These observations suggest that non-bCSC presented a higher level of origin firing, compared with bCSCs, potentially compensating numerous stalled replication forks. To confirm this hypothesis, we measured replication-fork progression using DNA-fiber analysis (Fig. 1E). Non-bCSCs showed reduced replication-fork speed compared with bCSCs, suggesting increased fork stalling. Altogether, these observations demonstrated that the RS is limited in the bCSC subpopulation compared with non-bCSCs. To evaluate the clinical relevance of the above findings, we scored a series of 30 breast tumor samples for the proportion of ALDH1-positive cells with γH2AX foci (Fig. 1F). Among the 30 tumor samples, only 2.4% of ALDH1-positive cells were also harboring γH2AX foci compared with 17.2% in the adjacent ALDH1-negative cells (*p* < 0.01) (Fig. 1G). This observation further validates that among clinical samples, bCSCs present a limited level of RS. To complete our vision of the RS sensing in bCSC, we evaluate the expression level of genes involved in RS response (ATR-dependent) [21]. We took advantage of the gene-expression profiling we have previously generated in bCSCs and non-bCSCs isolated from 8 different PDX models [9] and performed a metagene analysis for the global expression of genes composing the RS-response signature. We observed a significant higher RS score in bCSC compared with non-bCSC (*p* < 0.0001) (Fig. 1H). This result suggests a proficient RS response in bCSCs that may explain the subsequent limited RS level in this cell subpopulation.

### Replicative bCSCs have enhanced activation of homologous recombination (HR)

**Fig. 2:**
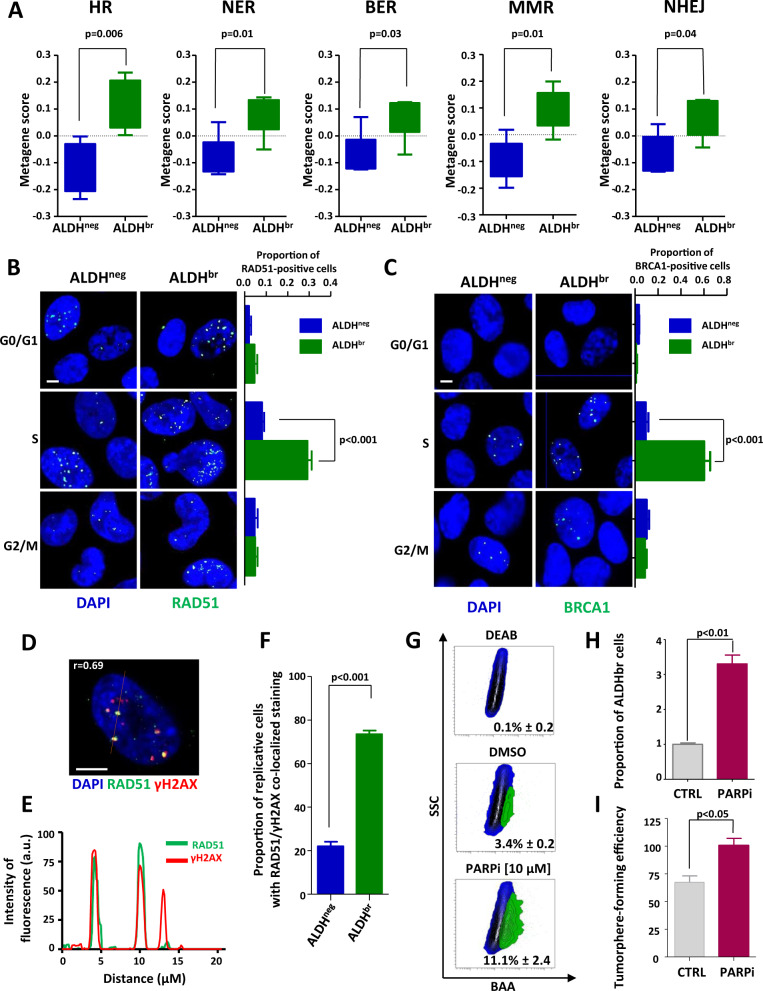
Replicative ALDH^br^ bCSCs demonstrated enhanced DNA-repair activity. **A** Box plots representing the gene-expression level of DNA-repair pathway metagenes in ALDH^br^ and ALDH^neg^ cells isolated from PDXs. **B**, **C** Representative images of RAD51 foci (**B**) and BRCA1 foci (**C**) (green staining) in ALDH^br^ and ALDH^neg^ cells sorted according to their cell cycle phase (G0/G1, S, and G2/M). Nuclei are counterstained with DAPI (blue staining). Scale bar: 5μm. Bar plots represent the proportion of RAD51-positive cells (**B**) or BRCA1-positive cells (**C**) for each cell subpopulation. **D** Representative images of RAD51 costaining (green foci) with γH2AX (red foci). Nuclei are counterstained with DAPI (blue staining). The red line corresponds to the line scan. Pearson’s coefficient evaluated the amount of colocalization. Scale bar: 5 μm. **E** Line-scan profile of the relative intensity of RAD51 and γH2AX fluorescent signals. **F** Bar plot representing the proportion of replicative cells with RAD51/γH2AX colocalized in ALDH^br^ and ALDH^neg^ SUM159 cells. **G** Representative examples of flow chart for the ALDEFLUOR staining following PARPi treatment in SUM159 cells. DEAB is an ALDH inhibitor used as negative control. **H** Bar plot representing the proportion of ALDH^br^ cells following PARPi treatment compared with the untreated condition (CTRL). **I** Bar plot representing tumorsphere-forming efficiency (SFE) for SUM159 cells under PARPi-treated and -untreated conditions. Statistical test used is Student’s *t*-test. Data represent mean ± SD.

Evidence of increased expression of many DNA-repair genes in CSCs [9, 10, 12, 13] led us to hypothesize that DNA-damage response (DDR) plays a key role in limiting RS in bCSCs. To better characterize which DNA-repair processes are activated in bCSCs, we constructed metagenes for the five major DDR pathways. Applying these metagene scores to bCSC and non-bCSC gene-expression profiles generated from PDXs, we observed a significant higher expression of all DNA-repair metagenes in bCSC compared with non-bCSCs (Fig. 2A). We confirmed the overexpression of different DNA-repair players in the bCSC population at the protein level (Supplementary Fig. 2A). Homologous-recombination (HR) metagene presented the more convincing differential score between both cell subpopulations (*p* = 0.006) with an upregulation in bCSCs of all key players such as RAD51, BRCA1, or MRE11 (Supplementary Fig. 2B). Due to the preponderant role of HR and particularly RAD51 to promote stressed replication-fork reversal [22, 23], we evaluate the proportion of replicative bCSCs and non-bCSCs with RAD51 or BRCA1 foci. bCSCs presented a significant increase of RAD51- and BRCA1-positive cells in S phase (Fig. 2B, C). Conversely, non-bCSCs showed a weak and steady proportion of RAD51- and BRCA1-positive cells in all cell-cycle stages. To further address the contribution of RAD51 in limiting RS of bCSCs, we performed co-immunostaining of RAD51 and γH2AX in replicative cells. We did observe a significant colocalization of RAD51 foci with γH2AX foci in replicative bCSCs (*r* = 0.69) compared with replicative non-bCSCs (*p* < 0.001) (Fig. 2D–F). This observation is consistent with the known recruitment of RAD51 to stressed replication forks [22, 24]. To further demonstrate HR overactivation in bCSCs, we exploited the concept of synthetic lethality using PARP inhibitor (PARPi). Hence, it has been demonstrated that only HR-proficient cells can survive spontaneous DNA single-strand breaks accumulated following PARP inhibition [25, 26]. PARP inhibition induced a 3.4-fold increase of ALDH^br^ cells’ proportion (Fig. 2G–H) and an increase in tumorsphere-forming efficiency (Fig. 2I), suggesting a strong selective induction of synthetic lethality in non-bCSCs.

Altogether, these results suggest that bCSCs are prone to activate HR during DNA replication, whereas non-bCSCs seem to present at least a partial HR deficiency.

### BMI1 nuclear distribution regulates RAD51-dependent response to replication stress

**Fig. 3:**
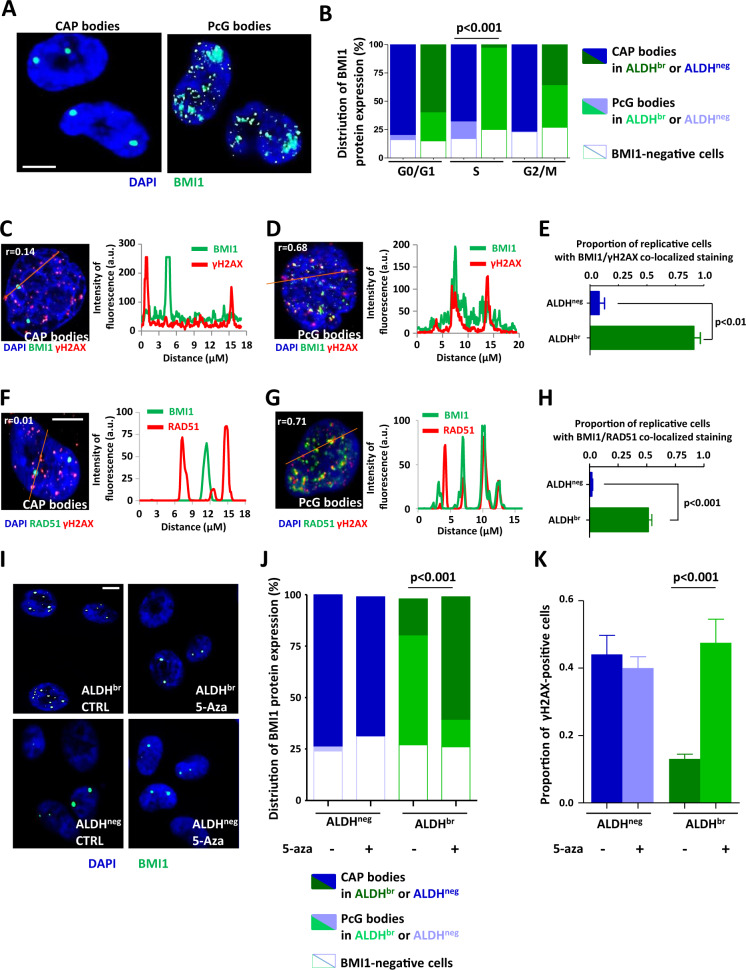
BMI1 nuclear location affects replicative stress response in breast cancer cells. **A** Representative images of BMI1 staining (green bodies). Nuclei are counterstained with DAPi (blue staining). BMI1 protein can accumulate in CAP bodies (left panel) or has uniform punctate distribution (PcG bodies, right panel). Scale bar: 5 μm. **B** Stacked bar plot representing the proportion of CAP and PcG bodies in ALDH^br^ and ALDH^neg^ SUM159 cells sorted according to their cell-cycle phase (G0/G1, S, and G2/M). **C**, **D** Representative images (left panels) of BMI1 costaining (green foci) with γH2AX (red foci) for cells harboring CAP bodies (**C**) or PcG bodies (**D**). Nuclei are counterstained with DAPi (blue staining). The red lines correspond to the line scans. Pearson’s coefficient evaluated the amount of colocalization. Scale bar: 5 μm. On the right panels, line-scan profiles of the relative intensity of BMI1 and γH2AX fluorescent signals. **E** Bar plots representing the proportion of replicative ALDH^br^ and ALDH^neg^ SUM159 cells with BMI1/γH2AX colocalization. **F**, **G** Representative images (left panels) of BMI1 costaining (green foci) with RAD51 (red foci) for cells harboring CAP bodies (**F**) or PcG bodies (**G**). Nuclei are counterstained with DAPi (blue staining). The red lines correspond to the line scans. Pearson’s coefficient evaluated the amount of colocalization. Scale bar: 5 μm. On the right panels, line-scan profiles of the relative intensity of BMI1 and RAD51 fluorescent signals. **H** Bar plots representing the proportion of replicative ALDH^br^ and ALDH^neg^ SUM159 cells with BMI1/RAD51 colocalization. **I** Representative images of BMI1 staining (green bodies) in ALDH^br^ and ALDH^neg^ SUM159 cells treated with 5-aza or untreated (CTRL). Nuclei are counterstained with DAPi (blue staining). **J** Stacked bar plot representing the proportion of CAP and PcG bodies in replicative ALDH^br^ and ALDH^neg^ SUM159 cells treated with 5-aza or untreated. **K** Bar plots representing the proportion of γH2AX-positive cells for each cell subpopulation (replicative ALDH^br^ and ALDH^neg^ SUM159 cells) following 5-aza treatment or in untreated conditions (CTRL). Statistical test used is Student’s *t*-test. Data represent mean ± SD.

We then whiled to investigate how HR is preferentially activated in bCSC. Several reports have demonstrated that numerous polycomb-group (PcG) proteins, including BMI1, are involved in HR activation [27, 28]. Based on the well-established role of BMI1 in regulating self-renewal of stem cells [29–32], we hypothesized that BMI1 could orchestrate RS response in bCSCs. As previously described in colorectal CSCs [29], BMI1 presented a similar expression in both cell fractions enriched for bCSCs and non-bCSCs (Supplementary Fig. 3A, B). Interestingly, subsequent characterization of BMI1 cellular localization revealed different patterns between bCSCs and non-bCSCs. We observed essentially two large nuclear BMI1 “bodies” in non-bCSCs. These large conglomerations of BMI1 proteins are known as “cancer-associated polycomb” bodies (CAP bodies) (Fig. 3A). They correspond to an aberrant PRC1 aggregation on the 1q12 megasatellite [33]. Costaining of BMI1 with CBX4 (another PRC1 component) further confirmed that these bodies are PRC1 aggregates (Supplementary Fig. 3C, D). Inversely, in bCSCs we mainly observed small, numerous, and widely distributed BMI1 foci (Fig. 3A). Using BMI1/CBX4 costaining, we observed a colocalization of these two PcG proteins, suggesting that these small BMI1 puncta could correspond to structures known as “PcG bodies” [34, 35] (Supplementary Fig. 3E, F). These PcG bodies were preferentially detected in replicative bCSCs (Fig. 3B). BMI1 recruitment to the DNA damage sites is among the earliest detectable response to DNA lesions [36]. Thus, we hypothesized that PcG bodies correspond to BMI1 recruitment to stressed replication forks in order to initiate RAD51 function. We performed co-immunostaining for BMI1 and γH2AX in replicative cells and observed a total absence of colocalization of CAP bodies with γH2AX foci (Spearman’s *r* = 0.14) (Fig. 3C). In contrast, PcG bodies colocalized with γH2AX foci (Spearman’s *r* = 0.68) (Fig. 3D, E). Similar observations were done for BMI1 and RAD51 co-immunostaining (Fig. 3F–H). Altogether, these results suggest that BMI1 could be distributed on stressed replication forks of bCSCs where RAD51 is recruited. To functionally prove the role of the aberrant distribution of BMI1 on the replicative-stress response, we enforced CAP body formation in bCSCs. It has been described that mega-satellite demethylation sponges up PRC1 into CAP bodies [33]. Thus, we treated SUM159 cells with 5-aza-2′-deoxycytidine (5-aza) that has been shown to demethylate 1q12 megasatellite [37]. Remarkably, within 24 hours of 5-aza treatment, BMI1 was massively aggregated into CAP bodies in replicative bCSCs (Fig. 3I, J). This BMI1 aggregation in CAP bodies was accompanied by a strong induction of γH2AX foci in replicative bCSCs (Fig. 3K). These results further support that BMI1 compartmentalization within nuclear structures contributes to replicative-stress response in bCSCs.

### BMI1/RAD51 axis inhibition promotes RS in bCSCs

**Fig. 4:**
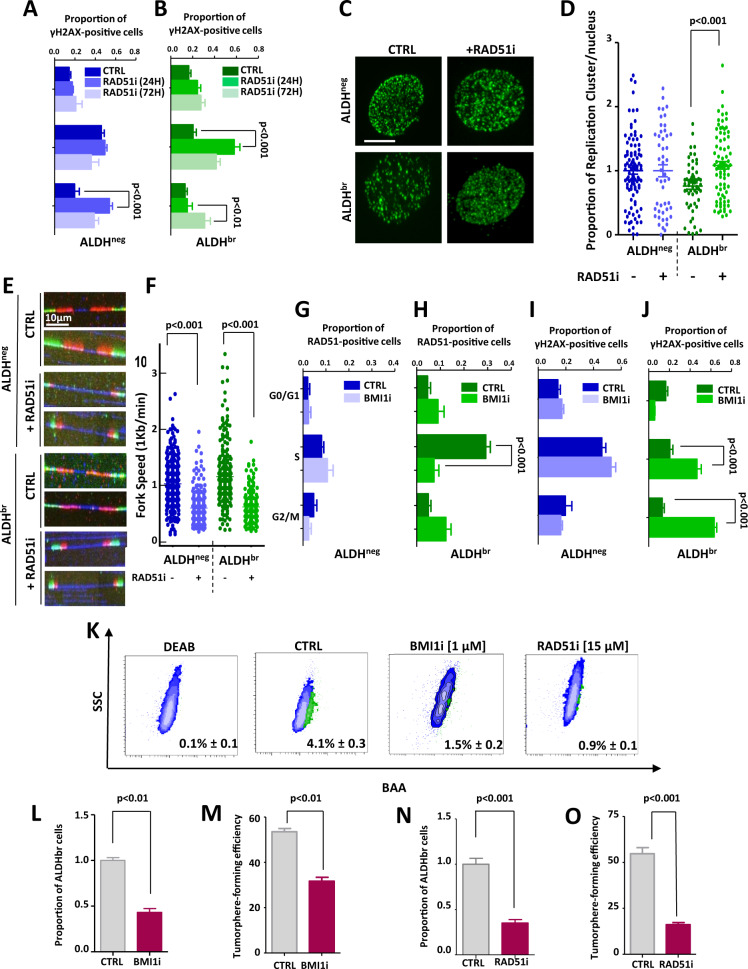
BMI1/RAD51 axis inhibition enhanced replication stress of ALDH^br^ bCSCs. **A**, **B** Bar plots representing the proportion of γH2AX foci in ALDH^neg^ (**A**) and ALDH^br^ (**B**) SUM159 cells sorted according to their cell-cycle phases, after 24 h/72 h of treatments with RAD51i or DMSO (CTRL). **C–F** Quantification of replication-stress markers in ALDH^br^ and ALDH^neg^ SUM159 cells treated with RAD51i or DMSO (CTRL). **C** Representative images of active replication clusters (green staining). Scale bar: 5 μm. **D** Bee-swarm plots representing the proportion of replication clusters per nucleus in each SUM159 cell subpopulation. **E** Representative images of combed DNA molecules. CldU (green staining) and IdU (red staining) were detected using specific antibodies and DNA is counterstained with DAPI (blue staining). Scale bar: 10 μm. **F** Bee-swarm plots representing the distribution of fork speed in each cell subpopulation. **G–J** Bar plots representing the proportion of RAD51-positive (**G**, **H**) or γH2AX-positive cells (**I**, **J**) in ALDH^neg^ (**G**, **I**) and ALDH^br^ (**H**, **J**) SUM159 cells sorted according to their cell-cycle phases, after treatment with BMI1i or DMSO (CTRL). **K** Representative examples of flowchart for the ALDEFLUOR staining following BMI1i or RAD51i treatment in SUM159 cells. DEAB is an ALDH inhibitor used as negative control. **L**, **N** Bar plot representing the proportion of ALDH^br^ cells following BMI1i (**L**) or RAD51i (**N**) treatment compared with the untreated condition (CTRL). **M**, **O** Bar plot representing tumorsphere-forming efficiency (SFE) for SUM159 cells under BMI1i (**M**) or RAD51i (**O**) treatment compared with untreated conditions (CTRL). Statistical test used is Student’s *t*-test. Data represent mean ± SD.

To address the contribution of BMI1/RAD51 axis in limiting RS of bCSCs we used two small-molecule inhibitors, one blocking RAD51 DNA binding (RAD51i known as B02) (Supplementary Fig. 4A, B) [38], and the other inhibiting BMI1 expression (BMI1i known as PTC209) (Supplementary Fig. 4C) [29]. Inhibition of RAD51 in bCSCs induced a strong increase of γH2AX-positive cells in S phase after 24 hours of treatment followed by an increase of γH2AX-positive cells in G2/M phase after 72 hours of treatment (Fig. 4A, B). This observation is concordant with a preponderant role of RAD51 in limiting RS in bCSCs, therefore preventing DNA-damage accumulation in post-replicative cells. Interestingly, for the non-bCSC population, RAD51 inhibition only increased the proportion of γH2AX-positive cells in G2/M phase (Fig. 4A). This result suggests that non-bCSCs conserved a restricted HR activity able to maintain solely a post-replicative DNA-repair function. We further confirmed in bCSCs that RAD51 inhibition increased the number of replicative foci and reduced replication-fork speed (Fig. 4C–F). These observations support a preponderant role of RAD51 in limiting RS in bCSCs. To evaluate the role of BMI1 in the regulation of RAD51-dependent response to replication stress, we treated cells with a BMI1i. We observed considerably reduced RAD51 foci in replicative bCSCs BMI1i-treated compared with untreated cells (Fig. 4G, H). This decrease of RAD51 foci was accompanied by an increase of γH2AX foci in replicative and post-replicative bCSCs BMI1i-treated (Fig. 4I, J). In contrast, no significant changes were observed for RAD51 and γH2AX foci distribution in non-bCSCs BMI1i-treated compared with untreated cells (Fig. 4G, I). A direct consequence of the RS induced by the BMI1/RAD51 axis inhibition is a significant decrease of the bCSC pool in treated cells, as assessed by a reduction of ALDHbr cells’ proportion and tumorsphere-forming efficiency (Fig. 4K–O). Similar results were observed with RNAi/shRNA constructs that interfere with RAD51 and BMI1 expression (Supplementary Fig. 4D–O). In conclusion, the BMI1/RAD51 axis is necessary to control and limit RS in bCSCs that appears to be selectively sensitive to an increased RS.

### BMI1/RAD51 axis prevents RS induced by cisplatin in bCSCs

**Fig. 5:**
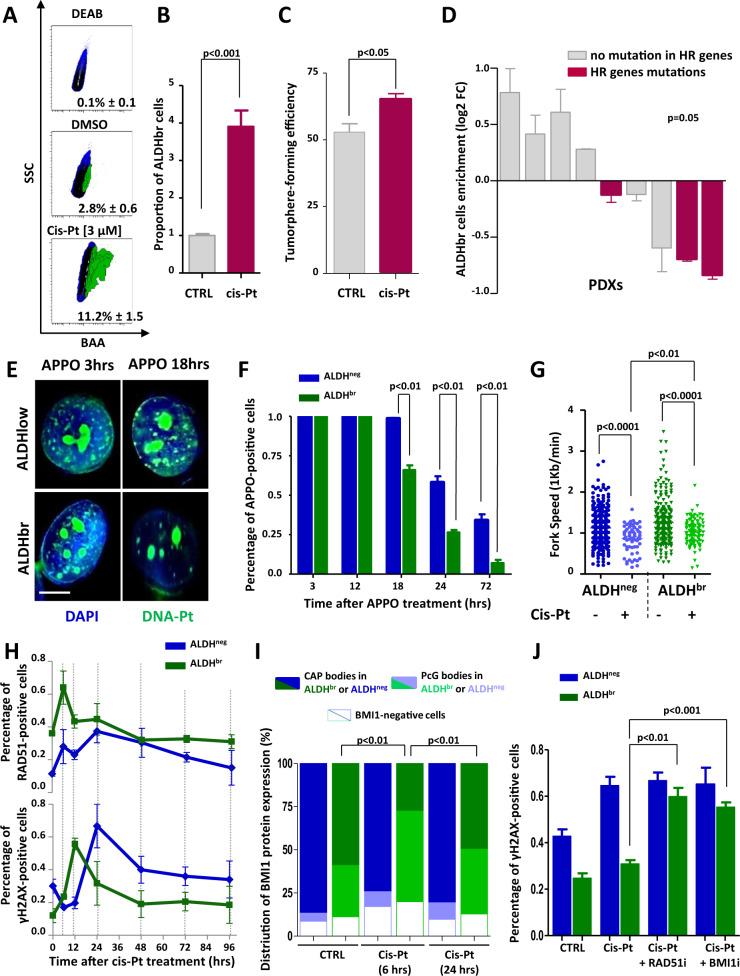
ALDH^br^ bCSCs resist to cisplatin in a BMI1/RAD51-dependent manner. **A** Representative examples of flowchart for the ALDEFLUOR staining following cisplatin treatment in SUM159 cells. DEAB is an ALDH inhibitor used as negative control. **B** Bar plot representing the proportion of ALDH^br^ cells following cisplatin treatment compared with the untreated condition (CTRL). **C** Bar plot representing tumorsphere-forming efficiency (SFE) for SUM159 cells under cisplatin-treated and -untreated conditions. **D** Waterfall plot representing the range of change in ALDHbr bCSC proportion in PDXs treated with cisplatin. Positive changes represent enrichment in bCSCs and negative changes in reduction of bCSCs compared with the untreated conditions. A two-sided chi-square test evaluates the probability to predict bCSC response to cisplatin according to the presence of HR gene mutation in PDX. **E** Detection of labeled DNA–pt lesions (green staining) in ALDH^br^ and ALDH^neg^ SUM159 cells using clickable cisplatin derivative APPO. Nuclei are counterstained with DAPI (blue staining). Scale bar: 5 μm. **F** Bar plot representing the proportion of ALDH^br^ and ALDH^neg^ SUM159 cells with DNA-pt lesions at different time points following APPO treatment. **G** Bee swarm plots representing the distribution of forks speed in each cell subpopulations untreated or treated with cisplatin. **H** Kinetic curves tracing the proportion of RAD51-positive or γH2AX-positive cells following cisplatin treatment, in each cell subpopulations. **I** Stacked bar plot representing the proportion of CAP and PcG bodies in ALDH^br^ and ALDH^neg^ SUM159 cells treated with 6 h/24 h of cisplatin compared with untreated condition (CTRL). **J** Bar plot representing the proportion of γH2AX-positive cells in ALDH^br^ and ALDH^neg^ SUM159 cells treated with cisplatin alone or in combination with RAD51i or BMI1i. Statistical test used is Student’s *t*-test. Data represent mean ± SD.

Because a large proportion of the therapeutic arsenal used to treat breast cancers ultimately acts through an enhancement of RS, we hypothesized that bCSCs resist to conventional therapies by lowering RS induced by the treatment. We first confirmed that using a genotoxic agent increasing replicative stress such as cisplatin, we induced an increase of the bCSC populations in SUM159 cells (Fig. 5A–C). Then, we treated with cisplatin a set of 9 independent PDXs maintained in a short-term culture assay. After three days of treatment, we observed an enrichment in ALDH^br^ cells in only four PDXs (Fig. 5D). However, this enrichment was significantly associated with the absence of alterations in genes involved in HR (Fig. 5D, Supplementary Fig. 5A, B). These observations suggest that HR could participate to bCSC cisplatin resistance. To further characterize the ability of bCSC to resist to cisplatin, we monitored the processing of toxic-induced DNA–Pt lesions (iDNA–Pt) generated by covalent bonds between this drug and purine residues [39]. We used a clickable cisplatin derivative, APPO [40], to detect in situ iDNA–Pt lesions at a single cell level. Concordant with previous observations [40], labeled iDNA–Pt exhibited spotted nuclear staining reflecting the targeting of nuclear DNA, and nucleoli staining corresponding to Pt drugs binding to rRNA (Fig. 5E). After 12 hours of treatment, both bCSCs and non-bCSCs presented a similar proportion of cells presenting iDNA–Pt lesions. Then, bCSCs progressively reduced the proportion of iDNA–Pt lesions with a greater kinetics than non-bCSCs (Fig. 5F). In addition, both cell subpopulations presented a reduced fork speed in cisplatin-treated condition compared with untreated cells (Fig. 5G). However, bCSCs maintained a higher fork speed compared with non-bCSCs under cisplatin treatment, suggesting their greater ability to respond to RS. Collectively, these data indicate that bCSCs tackle more efficiently DNA lesions and RS generated by genotoxic treatment than non-bCSCs. To determine whether the preponderant activation of the BMI1/RAD51 axis in bCSCs could contribute to the cisplatin resistance, we monitored the co-evolution of RAD51, BMI1, and γH2AX foci in treated cells. bCSCs presented a rapid and transient formation of RAD51 foci after 6 hours of treatment followed by a rapid onset of γH2AX foci after 12 hours of treatment (Fig. 5H), while non-bCSCs accumulate a significant amount of γH2AX foci after 24 hours of treatment with a moderate change in the proportion of RAD51 foci. Similar observations were made for BMI1 staining with the rapid and transient formation of typical nuclear PcG bodies in bCSCs after 6 hours of treatment, whereas non-bCSCs presented an elevated and constant proportion of cells with CAP bodies (Fig. 5I). These observations suggest a rapid BMI1/RAD51 recruitment in order to induce repair of iDNA–Pt lesions in bCSCs. To functionally test this hypothesis, we first cotreated cells with RAD51i and cisplatin and measured γH2AX foci in replicative cells. After 24 hours of treatment, the proportion of bCSCs with γH2AX foci was significantly increased in cotreated cells compared with cisplatin-treated cells (Fig. 5J). Consistently, cotreatment with BMI1i and cisplatin leads to similar observations with a significant increase of bCSCs with γH2AX foci compared with cisplatin-treated cells (Fig. 5J). Interestingly, BMI1 or RAD51 inhibition seems to sensitize bCSCs to cisplatin as shown by a reduction of the ALDH^br^ cell proportion in the RAD51i/cisplatin-cotreated cells and a reduction in the BMI1i/cisplatin conditions compared with the cisplatin-treated cells (Supplementary Fig. 5C, D).

### RAD51 inhibition sensitizes bCSC to cisplatin

**Fig. 6:**
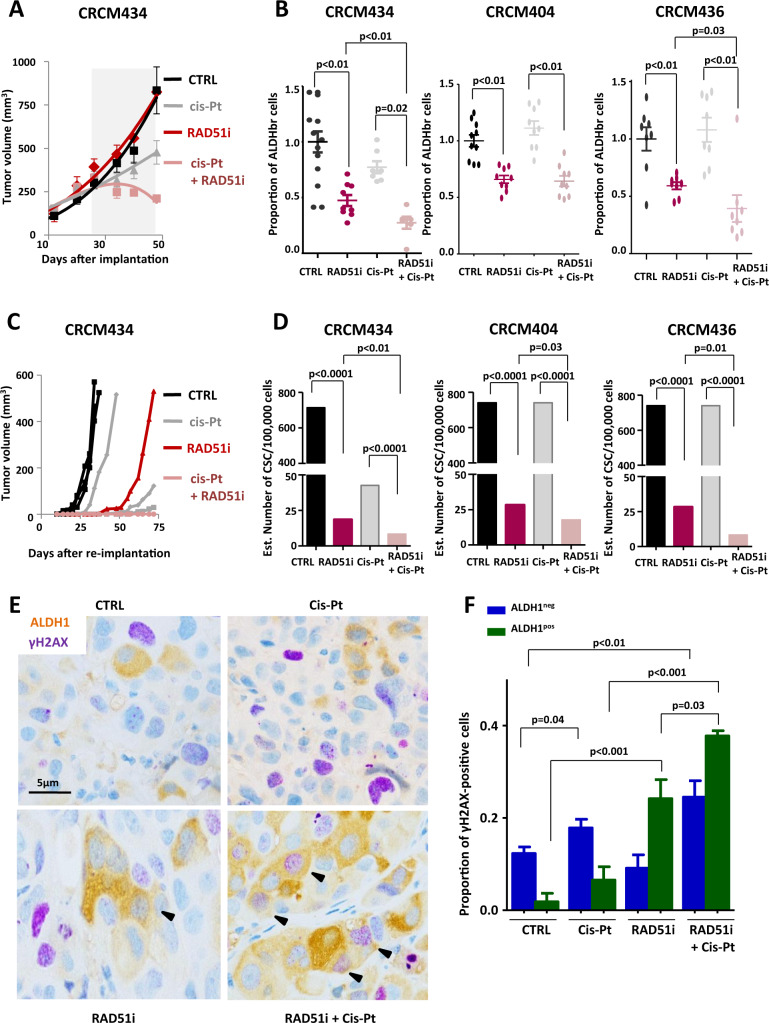
Cisplatin/RAD51i combination decreases the bCSC population in PDXs through an enhanced replication stress. **A** Effect of RAD51i and cisplatin treatment alone or in combination on the tumor growth of CRCM434, compared with the vehicle-treated condition. The gray area corresponds to the treatment period. **B** Bee-swarm plots representing the proportion of ALDH^br^ bCSCs in treated PDXs (CRCM434, CRCM404, and CRCM436) compared with the proportion of ALDH^br^ bCSCs in the vehicle‐treated tumors. **C** Reimplantation assay (CRCM434). Three-week treated PDXs were reimplanted, in serial dilutions, into new recipient mice, and tumor growth was monitored. Each curve represents the growth kinetics from one individual injection. **D** Bar plots representing bCSC frequency calculated using an extreme limiting dilution analysis (ELDA). The results are expressed as the estimated number of bCSCs for 100,000 tumor cells. Statistical test used is pairwise chi-square test. **E** Representative images of tumor section from PDX treated with RAD51i and cisplatin alone or in combination, compared with the vehicle-treated tumors (CTRL). Each section was co-immunostained with γH2AX (purple staining) and ALDH (brown staining). Arrowheads point out to costained cells. Scale bar: 5 μm. **F** Bar plot representing the proportion of γH2AX-positive cells in ALDH1^pos^ and ALDH1^neg^ cell subpopulations detected by co-immunostaining on PDX-treated tumor sections. Statistical test used is Student’s *t*-test. Data represent mean ± SD.

To validate RAD51 inhibition as an appropriate strategy to sensitize bCSCs to treatment such as cisplatin, we performed a preclinical assay using three breast cancer PDXs (CRCM434, CRCM404, and CRCM436) (Supplementary Fig. 6A). We did not observe any systemic toxicity for each treatment condition (Supplementary Fig. 6B). RAD51i had no effect on PDX growth at least in the timeframe of our experiments (Fig. 6A, Supplementary Fig. 6C). These observations are concordant with previous reports on anti-bCSC therapy that selectively targets bCSCs while mainly sparing actively dividing differentiated cancer cells, ending up with limited short-term effect on tumor growth [41–43]. Cisplatin alone or in combination with RAD51i reduced tumor growth of CRCM434 and CRCM404 without affecting CRCM436 tumor growth (Fig. 6A, Supplementary Fig. 6C). Concordantly, these observations are associated with an increase of apoptotic cells in treated tumors without any impact on cell proliferation (Supplementary Fig. 6D, E). To detect a potential impact on the tumorigenic cell populations, we first measured the proportion of ALDH^br^ cells after 3 weeks of treatment. PDXs treated with cisplatin did not show any significant changes of the ALDH^br^ cell proportion, whereas RAD51i treatment induced a decrease of the ALDH^br^ cell population in all PDX models (Fig. 6B). Moreover, we observed a pronounced decrease of the ALDH^br^ cell population in tumor cotreated with RAD51i and cisplatin. To functionally prove the reduction of the tumorigenic cell population in the treated tumors, we performed a limiting dilution transplantation assay into secondary mice (Supplementary Fig. 6G). For all PDX models, residual cells isolated from RAD51i-treated tumors presented a reduction of the tumor-initiating capacity in secondary mice compared with control and cisplatin (Fig. 6C, D, Supplementary Fig. 6F). In addition, residual cells isolated from the cotreated tumors presented the lower tumor-initiating capacity, indicating that bCSCs were sensitized to cisplatin. Interestingly, tumors treated with RAD51i presented an increase of ALDH1-positive cells presenting γH2AX foci (Fig. 6E, F). This observation was exacerbated in tumors cotreated with RAD51i and cisplatin. Concordantly, only cisplatin treatment, alone or in combination with RAD51i, induced an increase of γH2AX foci in the adjacent ALDH1-negative cells. Taken together, our data sustain that RAD51i treatment specifically sensitizes bCSCs to cisplatin and effectively controls tumorigenicity in a preclinical model of primary human breast cancer.

## Discussion

If replication stress (RS) can be considered as one of the main drivers of tumor initiation paradoxically in cancer cells with high genomic instability, it can be lethal, and may represent a dreadful therapeutic weapon. Thus, tumor cells best equipped for RS protection may be favored under genotoxic treatment. In this study, we demonstrated that breast CSCs, isolated from established tumors, managed to reduce replication alterations to duplicate their genome in stress conditions. Several studies performed in glioblastomas suggest that RS drives constitutive activation of the DNA-damage response in CSCs [44, 45]. Accordingly, we observed in breast cancer an activation of HR during CSC DNA replication. Moreover, RAD51 inhibition promotes a massive RS in bCSCs further demonstrating RAD51 pivotal role in preventing DNA damage accumulation in post-replicative bCSCs.

DNA-repair programs and more generally DNA-damage response (DDR) has been recurrently associated with stemness in normal and malignant tissues [14, 17]. Despite this observation, molecular mechanisms explaining the constitutive activation of DNA repair systems in CSCs remain elusive. Such robust association should reflect intertwined pathways coordinating CSC fate and DDR. Different chromatin regulators have been proposed to present this dual function. As an example, ZEB1, a major regulator of cancer-cell plasticity, has been identified as an ATM substrate. ATM phosphorylates and stabilizes ZEB1 that promotes homologous-recombination-dependent DNA repair via CHK1 [46]. At the same time, ZEB1 represses the highly mutagenic theta-mediated end joining (TMEJ) pathway to control the stability of ZEB1-positive cancer-cell genome [47]. Recently, SPT6, a histone chaperone, has been described to be essential for the DNA repair and maintenance of glioblastoma CSCs [48].

In our study, we demonstrated that BMI1 triggers the recruitment of RAD51 during DNA replication, limiting accumulation of DNA-damages in breast CSCs. Several reports clearly linked BMI1 and more generally PcG proteins (PRC1 and PRC2) to the response to DNA damages [49]. PRC1 and PRC2 proteins are rapidly recruited to sites of DNA damage. While PRC2 is thought to promote the recruitment of PRC1, the underlying mechanism remains unclear. Regarding PRC1, several reports described rapid PRC1 proteins (RING1A, RING1B, and BMI1) recruitment to DNA damage, further triggering the recruitment of BRCA1 and RAD51 [28]. These observations are concordant with our study showing that, in breast CSCs, BMI1 colocalized with γH2AX and RAD51 foci. Moreover, here we proposed that BMI1 and RAD51 are specifically recruited in bCSCs to stressed replication forks to limit RS and allow S-phase progression. These observations imply that BMI1 function is finely tuned between bCSC and non-bCSC. Because we did not observe any difference of BMI1 mRNA or protein expression between both cell subpopulations, we suspected that BMI1 activity in bCSCs was due to another process.

Hence, we identified a potential role of pericentric satellites in the regulation of BMI1 nuclear location during differential response to RS. We observed a redistribution of BMI1 proteins from CAP to PcG bodies in replicative breast CSCs but not in mature cancer cells. These CAP bodies are known as PRC1 aggregates on the high-copy satellite II (HSATII) sequences at the 1q12 megasatellite. HSATII acts as molecular sponges and sequesters chromatin-regulatory proteins such as BMI1 [33]. If HSATII demethylation appears to be the main mechanism explaining CAP body formation, regulators of these histone marks remain to be determined. In this context, it is striking to consider that pericentric satellites, initially defined as “junk” repeats, may play a preponderant role in the response of breast CSCs to RS, as a balance for BMI1 nuclear localization/function.

Constitutive activation of DNA-repair pathways in CSCs has a direct link with therapeutic resistance. It may confer the superior capacity of CSCs to evade DNA-damaging therapies. Bao et al. have pioneered this concept and demonstrated that radio-resistant glioblastoma cells are enriched in CSCs with an active DDR [10]. Recent reports confirm this seminal observation and demonstrated that glioblastoma CSCs are more efficient to repair radio-induced DNA breaks via both HR and NHEJ [45, 48]. Accordingly, we observed a superior capacity of bCSCs to resist to cisplatin due to a rapid processing of toxic iDNA–Pt lesions compared with mature cancer cells. Beyond the involvement of DNA-repair pathways in mediating the resistance of CSCs to DNA-damaging agents, it may also challenge the concept of synthetic lethality. Indeed, a subset of breast cancers presenting germline-mutated BRCA are currently treated with PARP inhibitors [25, 26]. If this strategy grandly improved PFS (patient-free survival) for the treatment of BRCA-mutated breast cancer, there is a growing prevalence of PARPi resistance [50]. One explanation may reside in the capacity of bCSCs from BRCA-mutated breast cancers to maintain a functional RAD51 activity avoiding the triggering of the synthetic lethality [51]. Current clinical trials propose to extend PARPi treatment to breast cancers with homologous-recombination deficiency (HRD). Based on these observations, HRD may be heterogenous in a tumor and it may be required to evaluate HRD in the bCSC subpopulation to predict response to PARPi.

Our findings of the role of BMI1/RAD51 axis in reducing RS and mediating therapeutic resistance in breast CSCs open new ways to treat cancer. Interestingly, we showed that inhibition of BMI1 or RAD51 induced a massive increase of RS in bCSC, ultimately leading to a decrease of the bCSC proportion. Similar observations have been done in colorectal and non-small-cell lung cancer with inhibition of CHK1 that increased the level of RS exclusively in CSCs, resulting in cell demise via replication catastrophe [12, 13, 52]. These observations support the concept that bCSCs are particularly vulnerable to increased RS and it may represent a new Achille heel for cancer treatment and prevention [53].

## Materials and methods

### Ethics statement

Samples of human origin and the associated data were obtained from the IPC/CRCM Tumour Bank that operates under authorization # AC-2013-1905 granted by the French Ministry of Research. Prior to scientific use of samples and data, patients were appropriately informed and filed a written consent, in compliance with French and European regulations. The experiments were conformed to the principles set out in the WMA Declaration of Helsinki and the Department of Health and Human Services Belmont Report. Animal studies were conducted in agreement with the French Guidelines for animal handling and approved by local ethics committee (Agreement no. #16487-2018082108541206 v3). Of note, mouse weight loss >20%, tumor necrosis, tumor volume >1500 mm3, ruffled coat + hunched back, weakness, and reduced motility were monitored daily and considered as endpoints.

### Cell culture

SUM159 was obtained from Dr. S. Ethier’s (Karmanos Cancer Center, Detroit, MI, USA), and was grown in standard medium as previously described [11]. Transformed SUM159 shCTRL and shRAD51 were given by Suling Liu’s laboratory (Key Laboratory of Breast Cancer, Shanghai, China) [51]. These cells were grown in the same medium than the SUM159 line but they received doxycycline (1/20,000, 10 mg/ml, Sigma).

### Breast cancer samples

Breast cancer samples were collected from a cohort of 108 consecutive patients with invasive breast cancers who underwent surgical biopsies or initial surgery at the Institut Paoli-Calmettes (Marseille, France) between 2014 and 2018. Among these 108 samples, we selected 30 for their positive expression for ALDH1.

### Drugs

BCLs were continuously treated for 72 h in adherent conditions with BO2 (RAD51i) (stock concentration SC = [10 mM], Sigma, SML0364), Cisplatin (SC = [10 mM], Selleckchem, S1166), Olaparib (PARPi) (SC = [10 mM], Selleckchem, S1060), PTC-209 (BMI1i) (SC = [10 mM], Selleckchem, S7372), 5-aza-2′-deoxycytidine (5-aza) (SC = [10 mM], Sigma, A3656), or APPO (SC = [10 mM], from R. Rodriguez’s laboratory, Institut curie, France) [40]. BO2, PTC-209, and Olaparib were resuspended in dimethyl sulfoxide (DMSO, Sigma), APPO was resuspended in DMF, and cisplatin in PBS. For the in vivo experiments, BO2 (SC = [5 mg/ml]) was resuspended in a solution of DMSO/cremophor 20% PBS, PTC-209 (SC = [32 mg/ml]) was resuspended in a solution of DMSO/PEG400, and cisplatin (SC = [150 mg/ml]) was resuspended in PBS.

### ALDEFLUOR assay

The analysis was processed on single‐cell suspension from cell lines or PDXs. The ALDEFLUOR Kit (Stem Cell Technologies) was used to isolate population with differential aldehyde-dehydrogenase enzymatic activity and analyzed using an LSR2 cytometer (Becton Dickinson Biosciences) as previously described [6]. To eliminate cells of mouse origin in PDX cell suspension, we used staining with an anti‐H2Kd antibody (#553563, BD Biosciences, 1:200, 20 min on ice). To isolate ALDH^br^/ALDH^neg^ cells according to their cell-cycle phase, we needed to perform DNA staining in living cells with a functional enzymatic activity. Thus, we used a cell-permeable DNA dye (Vybrant) with a low cytotoxicity. Cells were simultaneously labeled with ALDEFLUOR dye and the Vybrant Dye Cycle Ruby stain (V10273, Thermofisher Scientific, final [stain] = 5 μM) for 35 min at 37 °C. Cell-cycle analysis and cell sorting were performed by flow cytometry.

### Cell viability and proliferation assays

Inhibitory concentration 50 (IC50), in SUM159, was evaluated using 3‐(4,5‐dimethylthiazol‐2‐yl)‐5‐(3‐carboxymethoxyphenyl)‐2‐(4‐sulfophenyl)‐2H‐tetrazolium (MTS) assay (Promega). BCLs were plated in adherent conditions in 96‐well plates at 5000 cells per well, except for SUM159 plated at 3000 cells per well. After 24 h, treatment with serial dilutions of drugs was started. The effect of treatment on cell viability was estimated after 72 h by addition of 100 μl of MTS solution (5 mg/ml in PBS) in each well. Cells were then incubated for 1 h at 37 °C. Absorbance was measured at 540 nm in a plate reader (Tecan). Absorbance in treated conditions was normalized with absorbance in control condition to determine concentration–response curves and approximate IC50 concentrations.

### Tumosphere assay

Cells were treated with drugs (using IC50) in adherent conditions for 72 h. Then, treated cells were plated in single‐cell suspension in 96‐well ultra‐low-attachment plates, in a serum‐free mammary epithelium basal medium [6]. The frequency of cancer cells with tumorsphere‐forming potential was determined using the Extreme Limiting Dilution Analysis by plating cells at 25/10/5/3/2 and 1 cell per well (*n* = 18–36 wells/condition). The number of wells containing at least one sphere after 10 days of culture was considered as positive.

### Immunofluorescence

After cell sorting, cells were cytospun and fixed with 4% paraformaldehyde for 10 min and permeabilized with 0.1% Triton X-100 for 5 min before blocking with protein block (Dako). Cells were labeled 1 hour at room temperature with anti-phospho-Histone H2AX (Ser139, clone JBW301, Merck Millipore 1/1000), anti-RAD51 (homemade mouse monoclonal antibody from Mauro Modesti (CRCM, France) 1:20,000), anti-BRCA1 (homemade mouse polyclonal antibody from Jean Feunteun (CNRS-UMR8200, France), anti-CBX4 (Cell Signaling, clone E6L7X, #30559, 1:200), and anti-BMI1-AlexaFluor 488 (Merck-Millipore, #05-637-AF488, 1:200). After 10 min of wash with TBST, BMI1 staining aside, cells were incubated for 30 min with anti-mouse (A-11029, ThermoFisher), 1:500) or anti-rabbit AlexaFluor 488 (A-11008, ThermoFisher, 1:500). DNA was counterstained with DAPI 4′,6-diamidino-2-phenylindole (Vectashield, Mounting Medium fluorescence with Dapi H-200). Images were acquired using epifluorescence microscope Leica and LSM880 confocal module operated by Zeiss Zen 2015 software (Carl Zeiss MicroImaging, Inc., Germany) using either a 40x (PlanApochromat, NA 1.3) or a 63X oil-immersion objective (PlanApochromat, NA 1.4). Cells with more than 8 foci for ɣH2AX, RAD51, or BRAC1 were considered as positive cells. For each condition, immunofluorescence scoring was done on 100 cells in three independent experiments.

### IDU staining

For replication-foci analysis, cells were labeled with IdU for 20 min. After cell sorting, cells were fixed, permeabilized, and DNA was denatured with 2 M HCl for 30 min before immunostaining with IdU primary antibody (Abcam, ab187742, 1:200) and anti-rat Alexafluor 488 secondary antibody (ThermoFisher Scientific, A11006, 1/500). *Z*-stack images (stack size 0.2 μm) were acquired on a Zeiss spinning-disk confocal microscope operated with Metamorph software using a 100X oil-immersion objective. In total, 50 nuclei were quantified per sample using Multi Target Tracing algorithm [20] and Matlab software.

### DNA molecular combing

Exponentially growing cells were pulse-labeled with 100UM Cldu and 100uM Idu for 30 min each. Labeled cells were harvested and sorted based on their ALDH enzymatic activity. In total, 20,000 cells of each population were included in agarose plug. DNA-fiber spreads were prepared with FiberPrep DNA extraction kit (Genomic Vision) and sent to DNA combing platform (Genomic Vision, France). This platform processed and spread the DNA fibers using the FiberComb^®^ system, an automated instrument that stretches individual DNA molecules on specially treated glass surfaces. The fibers were then incubated with primary monoclonal antibodies to bromoxyuridin and 5-iodo-2′-deoxyuridine. They were then labeled with AlexaFluor 555 and AlexaFluor 488 secondary antibodies. After combing, the slides were scanned by a FiberVision^®^ scanner to capture images. Analysis was performed with the FiberStudio® system. This software is designed to detect, measure, and interpret hybridization signals on combed DNA. From the digital slide, which represents the equivalent of thousands of microscopic fields, the software extracts the signals of interest, analyzes their length, and generates easily interpretable results. An average of 200 fibers in each condition were analyzed.

### Digital quantification

The line-scan function in the ImageJ analysis software was used to measure relative signal intensities for each channel of a 3-color digital image of cell nuclei. Line regions were drawn across the entire nucleus of individual cells and pixel intensity along the line measured. To determine colocalization between two different proteins’ foci distribution, we used JACoP ImageJ plug-in [54]. The amount of colocalization was evaluated with a Pearson’s coefficient.

### Cell transfection

We used siRNA gene silencing to specially target and knockdown BMI1 gene. siRNA (6442 S, Cell Signaling) was lipoplexed with Lipofectamine RNAiMAX (Life Technologies) in a 96-well culture plate or in a 100 mm tissue-culture dish. After 15 min of complexation, cells were seeded on top of the lipoplexes (3000 cells/well for 96 wells or 600,000 cells for petri dish, final [siRNA] = 25 nM), and incubated for two days at 37 °C and 5% CO2 in a humidified incubator. Cell transfection was normalized with scrambled siRNA (silencer negative control, AM4611, ThermoFisher)

### mRNA extraction and quantitative real-time RT-PCR

mRNAs were extracted from cultured cell lines using RNeasy mini kit (QIAGEN, 74104). About 5 ug of RNA was reverse transcribed in accordance with the manufacturer’s instruction (Superscript II reverse transcriptase, Invitrogen). RNA expression levels were quantified using TaqMan probes (RAD51, Hs00947967_m1) (GAPDH, Hs03929097_g1) (BRCA1, Hs01556193_m1) (BRCA2, Hs00609073_m1) (RAD52, Hs01028879_m1) (RAD54L, Hs00936482_m1) (RPA1, Hs00161419_m1). RNA GAPDH expression was used for data normalization. Fold-change expression was calculated using the 2 − ΔΔCt method.

### Immunoblot analysis

Cells were lysed in ice-cold lysis buffer containing Hepes 50 nM, pH 7.5, EDTA 1 mM, pH 7, NaCl 150 mM, Naf 100 mM, Na3VO4 1 mM, Triton X-100 1%, and Proteinase Inhibitor Cocktail. Cell lysates were migrated in 12% SDS-PAGE (Sodium Dodecyl Sulfate–PolyAcrylamide Gel Electrophoresis). The following primary antibodies were used: anti-RAD51 (1/1000), anti-BMI1 (#2830, Cell Signaling, 1/1000), anti-LIG4 (MA5-32775, Thermo, 1/1000), anti-ATR (sc1887, Santa Cruz, 1/1000), and anti-phospho-ATR (2853 s, Cell Signaling, 1/1000), anti-ERCC1 (sheep mAb, Gift from C. Lachaud (CRCM, Marseille), 1/1000), and anti-MLH1 (BD Biosciences, 1/1000). Detection of vinculin (V9131, Sigma, 1/2000) or GAPDH (Rabbit pAb, Cell Signaling, 1/5000) was used as loading control.

### Animal models

In this study, we utilized a panel of nine primary human breast cancer xenografts generated from nine different patients (CRCM168, CRCM226, CRCM348, CRCM389, CRCM392, CRCM404, CRCM434, CRCM436, and CRCM494). These patient-derived xenografts (PDXs) were generated from chemo-naive breast tumors [9]. To explore the response to cisplatin of our panel of PDXs, we performed short-term culture assay. Cells from each PDX were isolated following tumor dissociation [9]. We used anti-H2Kd antibody (BD Biosciences, 1:200, 20 min on ice) to eliminate cells of mouse origin from each single-cell suspension. Then, tumor cells were plated in suspension in a 96-well plate ultralow attachment (10,000 cells per well) in SUM159 standard medium and treated with serial dilutions of cisplatin. The effect of treatment on cell viability was estimated after 72 hours using alamarBlue assay (Thermofisher). Inhibitory concentration 50 (IC50) was evaluated for each PDX and used subsequently in a second short-term culture assay to determine the effect of cisplatin treatment on the ALDHbr cell proportion. We utilized three PDXs (CRCM494, CRCM434, and CRCM226) to perform preclinical assay in vivo. Cells from these PDXs were transplanted orthotopically into fat pads of NSG mice without cultivation in vitro. We injected 300,000 cells per fat pad of NSG mice (with two injected fat pads per mouse) and monitored tumor growth. When tumors reached an average size of 50–100 mm3, mice were randomized (*n* = 4, i.e., 8 tumors for each PDX and for each group) and used to determine the response to the treatment. We initiated treatment with BMI1i (i.p., 60 mg/Kg, 5 out of 7 days, 2 weeks), alone, RAD51i alone (i.p., 10 mg/Kg, every second day, 2 weeks), cisplatin alone (i.p., 3 mg/Kg, once a week, 2 weeks), RAD51i/cisplatin combination, or placebo injected with a solution of DMSO/cremophore 20% PBS or DMSO/PEG400. After 2 weeks of treatment, mice from each group were sacrificed according to ethic statements. Tumors were cut in two pieces, with one piece fixed in formalin and paraffin-embedded for further histological analysis and the second piece was dissociated into single cells. These cells were analyzed for the ALDEFLUOR phenotype and reimplanted into secondary NSG mice. We performed serial dilution to functionally evaluate the proportion of residual CSCs in each group of treatment (CTRL, BMI1i, RAD51i, cisplatin, and RAD51i + cisplatin) from the 3 different PDXs. Each mouse that presents a tumor reaching a size of 25 mm^3^ was considered as a tumor-bearing mouse.

### Mutation and copy-number detection

For each PDX, we identified molecular alterations by performing targeted next-generation sequencing (NGS) and array-comparative genomic hybridization (aCGH) as previously described [55]. aCGH was done using high-resolution 244 K CGH microarrays (Hu-244A, Agilent Technologies) and targeted NGS was applied to a custom-made panel of 365 “cancer-associated” genes (CCP-V6 panel [56]). To determine copy-number alterations in each PDX, we mapped all aCGH probes according to the hg19/NCBI human genome mapping database. The copy number was estimated for each gene by taking the value of the segment with the highest amplitude, then categorized into “Amplified” (Log2ratio > 1), “Gain” (0.5 < Log2ratio < =1), “Loss” (−1 < =Log2ratio < −0.3), and “deletion” (Log2ratio < −1). Focal events were defined as genomic alterations with a size less than 5 Mb and a copy number higher than the surrounding segments. The percentage of genome altered was calculated as the sum of altered probe divided by the total number of probes. To determine the mutation profile of each PDX, we combined two data analysis pipelines as described previously [55]. Briefly, the first pipeline used FreeBayes version 0.9.9 for single-nucleotide variant (SNV) calling and insertions/deletion (indel) calling was done using GATK haplotype caller version 2.5-gf57256b with default parameters. For the second pipeline SNV calling was done with Mutect 1.7 and somatic indel calling with scalpel. All variants were then annotated for genes and function using ANNOVAR (version 2013-11-12). In order to remove false positives, recurrent variants with no entry in public databases such as COSMIC or dbsnp were removed. Variants identified by both pipeline analyses were retained as somatic.

### Gene-expression profiling

Gene expression profiling of PDXs was done with Affymetrix U133 plus 2.0 human oligonucleotide microarrays as previously described [9]. The molecular subtypes of PDXs were determined using the PAM50 predictor [57]. Gene-expression data of bCSC/non-bCSC were generated previously on ALDHbr/ALDH-negative cells isolated from 8 PDX samples [9]. Meta-subnetworks generated from reactome pathway database (https://reactome.org) for RSR, HR, NER, BER, MMR, and NHEJ were evaluated at mRNA level using metagene-based approach with the respective gene lists. With a natural cutoff of 0, PDX samples were classified as high or low level for each of the selected meta-subnetworks

### Detection of DNA–Pt lesions

For DNA–Pt lesion detection, cells were treated with APPO (final [APPO] = 100 μM) and collected at different time points. Then, viable cells were sorted based on their ALDH enzymatic activity using ALDEFLUOR assay. Sorted cells were washed with PBS and preextracted with CSK buffer (10 mM Pipes, pH 7.0, 100 mM NaCl, 300 mM sucrose, 3 mM MgCl2, and 0.7% Triton X-100) twice for 3 mins. Then, cells were washed with PBS, cytospun and fixed with 4% PFA for 10 min, and permeabilized with Triton X-100 0,1% for 5 min. APPO labeling was done with Click-it reaction (C10269, ThermoFisher Scientific) and alkyne AlexaFluor 488 (A10267, ThermoFisher Scientific). DNA was counterstained with DAPI. For each condition, immunofluorescence scoring was done on 100 cells in three independent experiments.

### Immunostaining on tumor sections

Paraffin-embedded human breast tissue sections and PDX were deparaffinized in xylene and rehydrated in graded alcohol. Immunohistochemistry was performed on a Ventana Discovery XT biomarker platform. For Ki-67 and cleaved-caspase-3 staining, antigen enhancement was performed by incubating the sections in citrate buffer, pH 6 (Dakocytomation). Staining was done using Peroxidase histostain-Plus Kit (Zymed) according to the manufacturer’s protocol. Ki-67 antibody (#R626, RTU, Agilent) and cleaved-caspase 3 (#9661 S, RTU, Cell Signaling) was used. DAB (Zymed) was used as substrate for peroxidase. For the multiplexed immunostaining of ALDH1 and γH2AX, we start with the anti-ALDH1 (mAb clone 44, Becton Dickinson, 1/50) antibody followed with the anti-γH2AX antibody (mAb JBW301, Merck Millipore, 1/2000). Multiplex staining was performed using Discovery OmniMap Multimer HRP with DAB substrate (Ventana) for ALDH1 and Discovery Purple kit substrate (Ventana) for γH2AX. Slides were counterstained with hematoxylin (Ventana).

### Statistical analysis

Graphpad Prism 5.0 was used for data analysis and imaging. The results are presented as mean ± SD for at least three repeated independent experiments. The choice of the sample size was based on our previous studies using similar experimental procedures [6, 9, 41, 42]. To investigate associations among variables, univariate analyses were performed using nonparametric Wilcoxon rank-sum test, chi-squared test or Fisher’s exact test when appropriate. Statistical analysis took into account unequal variance and applied the Welsh degrees-of-freedom correction when using parametric analysis. Extreme limiting-dilution analysis (http://bioinf.wehi.edu.au/software/elda/) was used to evaluate breast CSC frequency. In all cases, a *p*-value < 0.05 was considered as statistically significant.

## Supplementary information

Supplemental Figures 1-6

Reproducibility Checklist

## Data Availability

The dataset supporting the conclusions of this article has been previously deposited in the European Genome-phenome Archive (EGA) repository: accession EGAS00001004554 and https://www.ebi.ac.uk/ega/home for t-NGS data and in the ArrayExpress database at EMBL-EBI under the E-MTAB-9998 accession number for array-CGH data.
