# Supplementary material for: BMI1 nuclear location is critical for RAD51-dependent response to replication stress and drives chemoresistance in breast cancer stem cells
Source: Cell Death Dis. 2022 Feb 2;13(2):96. doi: 10.1038/s41419-022-04538-w (PMC8811067; doi:10.1038/s41419-022-04538-w)
Supplement: Supplementary file 1 — Supplemental Figures 1-6 [file 41419_2022_4538_MOESM1_ESM.pdf]

Supplementary Figures

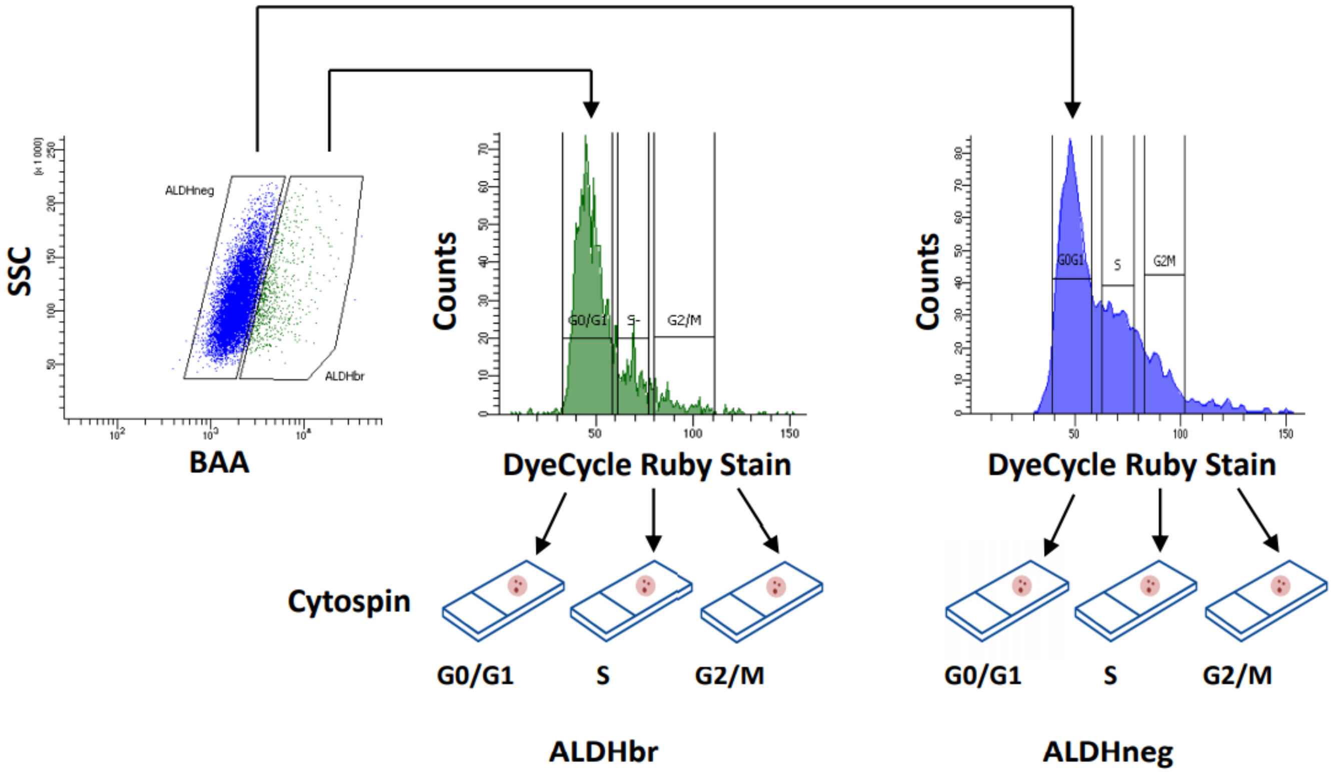

Supplementary Figure 1. Schematic representation of the experimental design used to sort ALDH^{br} and ALDH^{neg} cells in different cell cycle phases. We performed flow cytometry analysis with ALDEFLUOR assay to detect bCSC/non-bCSC and Vybrant Dye Cycle (Ruby stain) to detect DNA content. FACS-sorted cells were cytospun for further analysis, such as immunofluorescent staining.

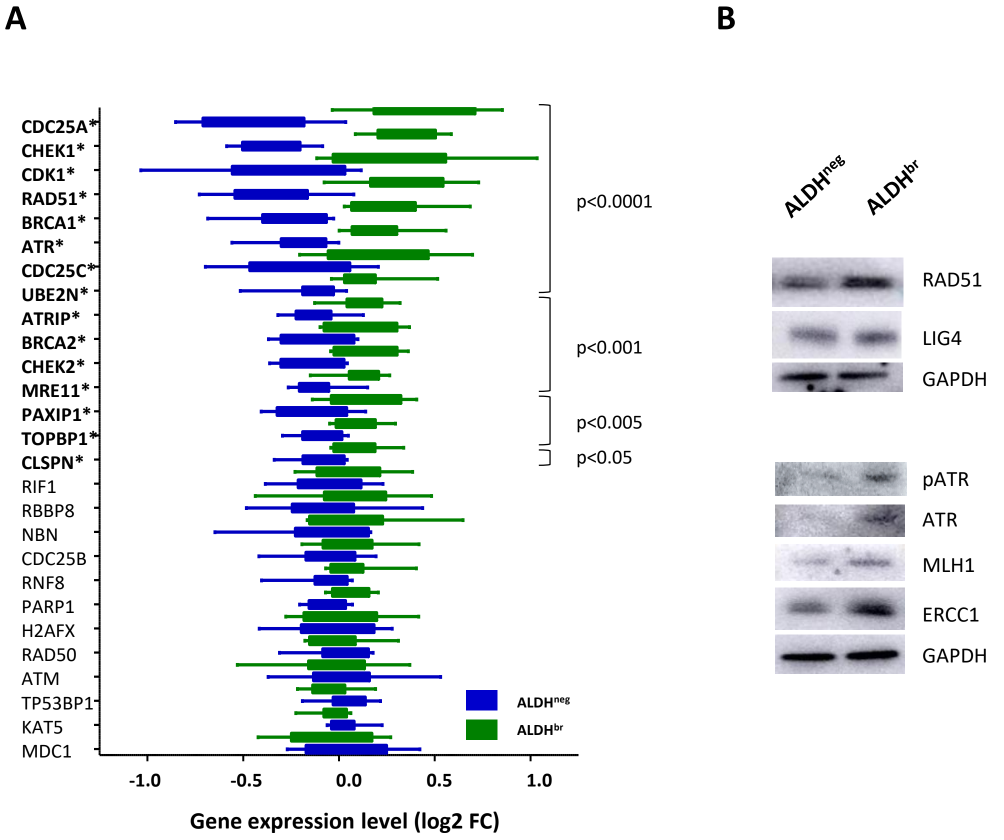

Supplementary Figure 2. Homologous recombination genes and protein expression in ALDHbr and ALDHneg cancer cells. A. Box plot representing expression level of genes from the homologous recombination machinery in ALDHbr and ALDHneg subpopulations isolated from PDXs. Genes are ranked according to their probability to be statically significant in its differential expression between both cell subpopulations. Statistical test used is Student's t-test. Data represent mean ± SD. **B.** RAD51, LIG4, pATR/ATR, MLH1, and ERCC1 proteins expression level evaluated in ALDHbr and ALDHneg SUM159 cells by western blot. GAPDH was used as a loading control.

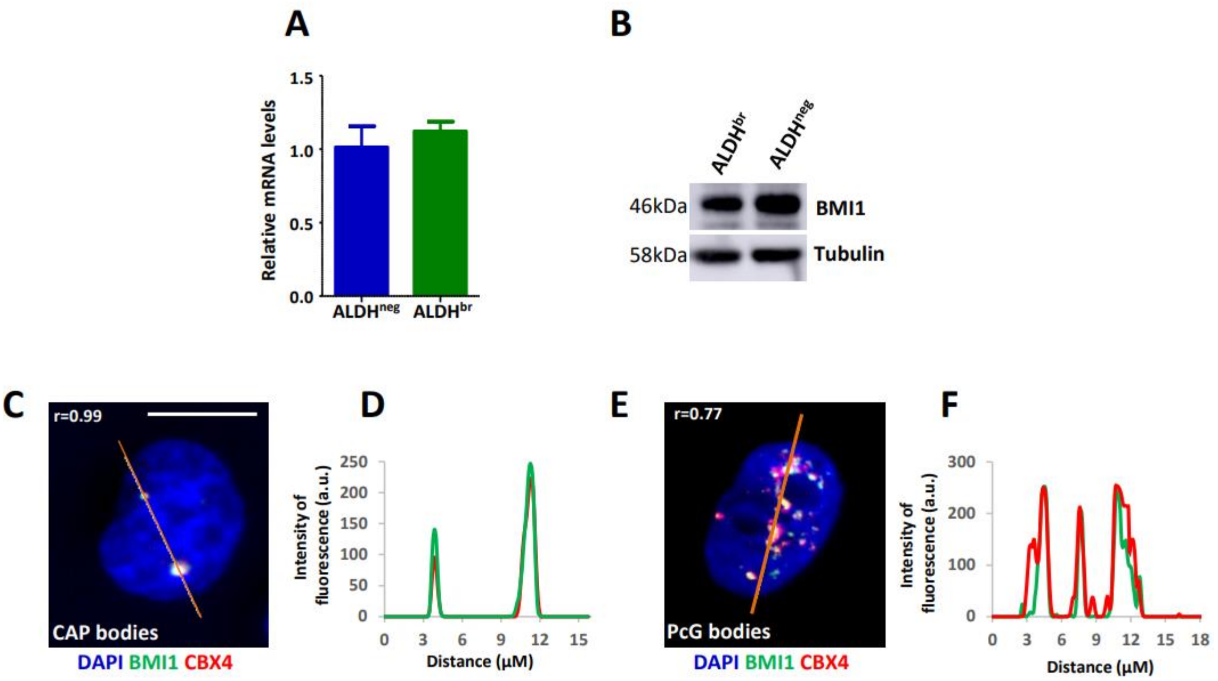

Supplementary Figure 3. BMI1 and CBX4 expression and nuclear location in ALDH^{br} and ALDH^{neg} cancer cells. Relative BMI1 expression in ALDH^{neg} and ALDH^{br} SUM159 cells was evaluated by RT-qPCR (**A**) and by western blot (**B**). Tubulin was used as a loading control. Representative images (left panels) of BMI1 co-staining (green foci) with CBX4 (red foci) for cells harboring CAP bodies (**C**) or PcG bodies (**D**). Nuclei are counterstained with DAPi (blue staining). The red lines correspond to the line scans. The Pearson's coefficient evaluated the amount of co-localization. Scale bar: 5µm. On the right panels, line scan profiles of relative intensity of BMI1 and CBX4 fluorescent signals.

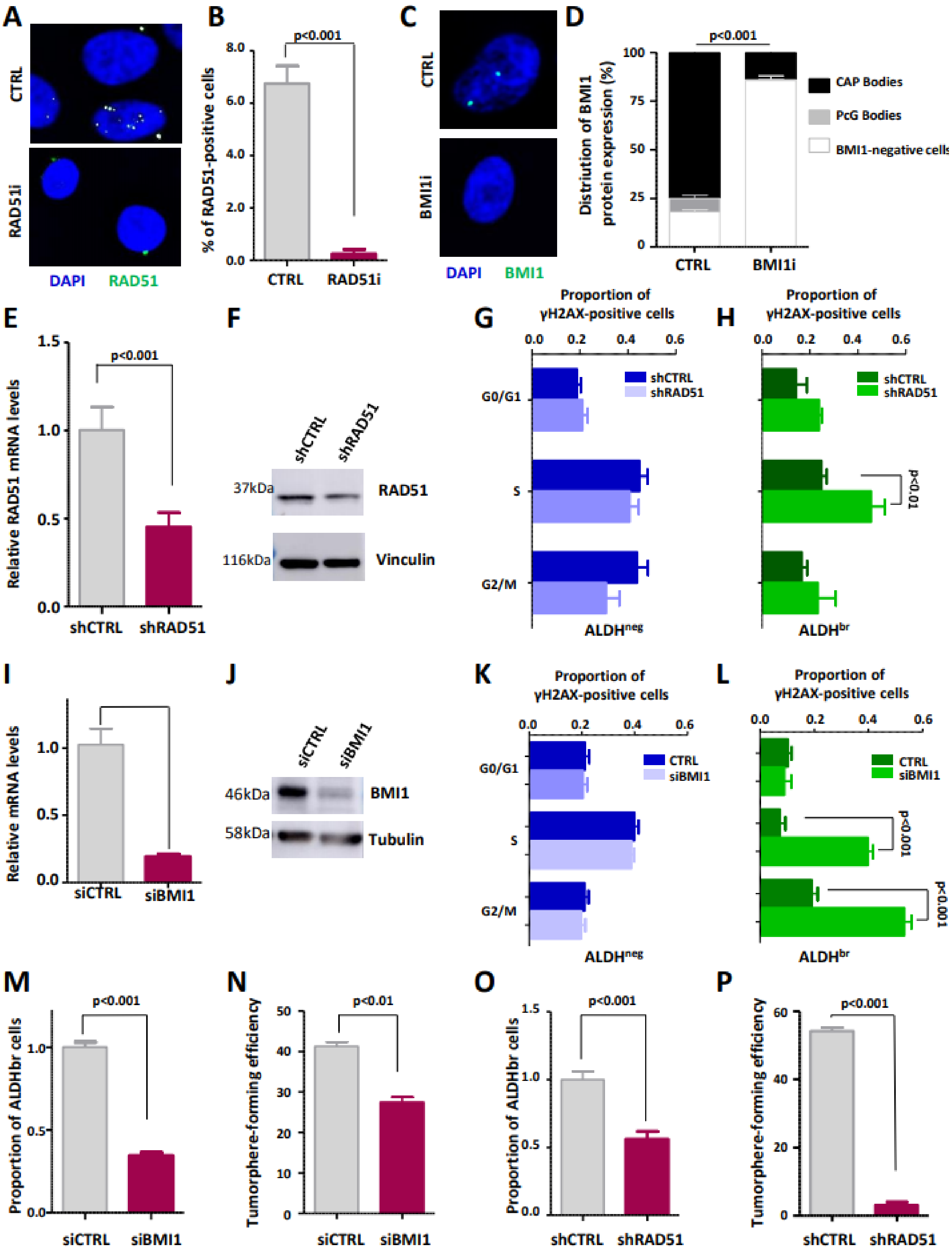

A
CTRL
RAD51i
DAPI RAD51
B
p<0.001
% of RAD51-positive cells
CTRL RAD51i
C
CTRL
BMI1i
DAPI BMI1
D
p<0.001
Distriution of BMI1 protein expression (%)
CAP Bodies
PcG Bodies
BMI1-negative cells
CTRL BMI1i
E
p<0.001
Relative RAD51 mRNA levels
shCTRL shRAD51
F
shCTRL shRAD51
37kDa RAD51
116kDa Vinculin
G
Proportion of γH2AX-positive cells
shCTRL
shRAD51
G0/G1
S
G2/M
ALDHneg
H
Proportion of γH2AX-positive cells
shCTRL
shRAD51
p<0.01
ALDHbr
I
Relative mRNA levels
siCTRL siBMI1
J
siCTRL siBMI1
46kDa BMI1
58kDa Tubulin
K
Proportion of γH2AX-positive cells
CTRL
siBMI1
G0/G1
S
G2/M
ALDHneg
L
Proportion of γH2AX-positive cells
CTRL
siBMI1
p<0.001
p<0.001
ALDHbr
M
p<0.001
Proportion of ALDHbr cells
siCTRL siBMI1
N
p<0.01
Tumorphere-forming efficiency
siCTRL siBMI1
O
p<0.001
Proportion of ALDHbr cells
shCTRL shRAD51
P
p<0.001
Tumorphere-forming efficiency
shCTRL shRAD51

Supplementary Figure 4. Effect of BMI1/RAD51 axis knockdown using a shRNA constructs on replication stress in ALDH^{br} bCSCs. A-D. Representative images (left panels) of RAD51 (**A**) or BMI1 staining (**C**) (green staining) after 24 hrs of treatment with RAD51i or BMI1i. Nuclei are counterstained with DAPi (blue staining). Scale bar: 5µm**.** On the right panels; bar plots representing the proportion of RAD51-positive (**B**) or BMI1-positive cells (**D**) after 24hrs of treatment with RAD51i or BMI1i. Statistical test used is Student's t-test. Data represent mean ± SD. **E-F.** RAD51 shRNA efficiency was evaluated by RT-qPCR (**E**) and by western blot (**F**). Vinculin was used as a loading control. **G-H.** Bar plots representing the proportion of γH2AX foci in ALDH^{neg} (**G**) and ALDH^{br} (**H**) SUM159 cells sorted according to their cell cycle phases, after RAD51 knockdown (shRAD51) or in control condition (shCTRL). **I-J**. BMI1 siRNA efficiency was evaluated by RT-qPCR (**I**) and by western blot (**J**). Tubulin was used as a loading control. **K-L.** Bar plots representing the proportion of γH2AX foci in ALDH^{neg} (left panel) and ALDH^{br} (right panel) SUM159 cells sorted according to their cell cycle phases, after BMI1 knockdown compared to control conditions. **M,O**. Bar plot representing the proportion of ALDH^{br} cells following BMI1 (**M**) or RAD51 (**O**) knockdown compared to the control condition. **N,P.** Bar plot representing tumorsphere-forming efficiency (SFE) for SUM159 cells following BMI1 (**N**) or RAD51 (**P**) knockdown compared to the control condition. Statistical test used is Student's t-test. Data represent mean ± SD.

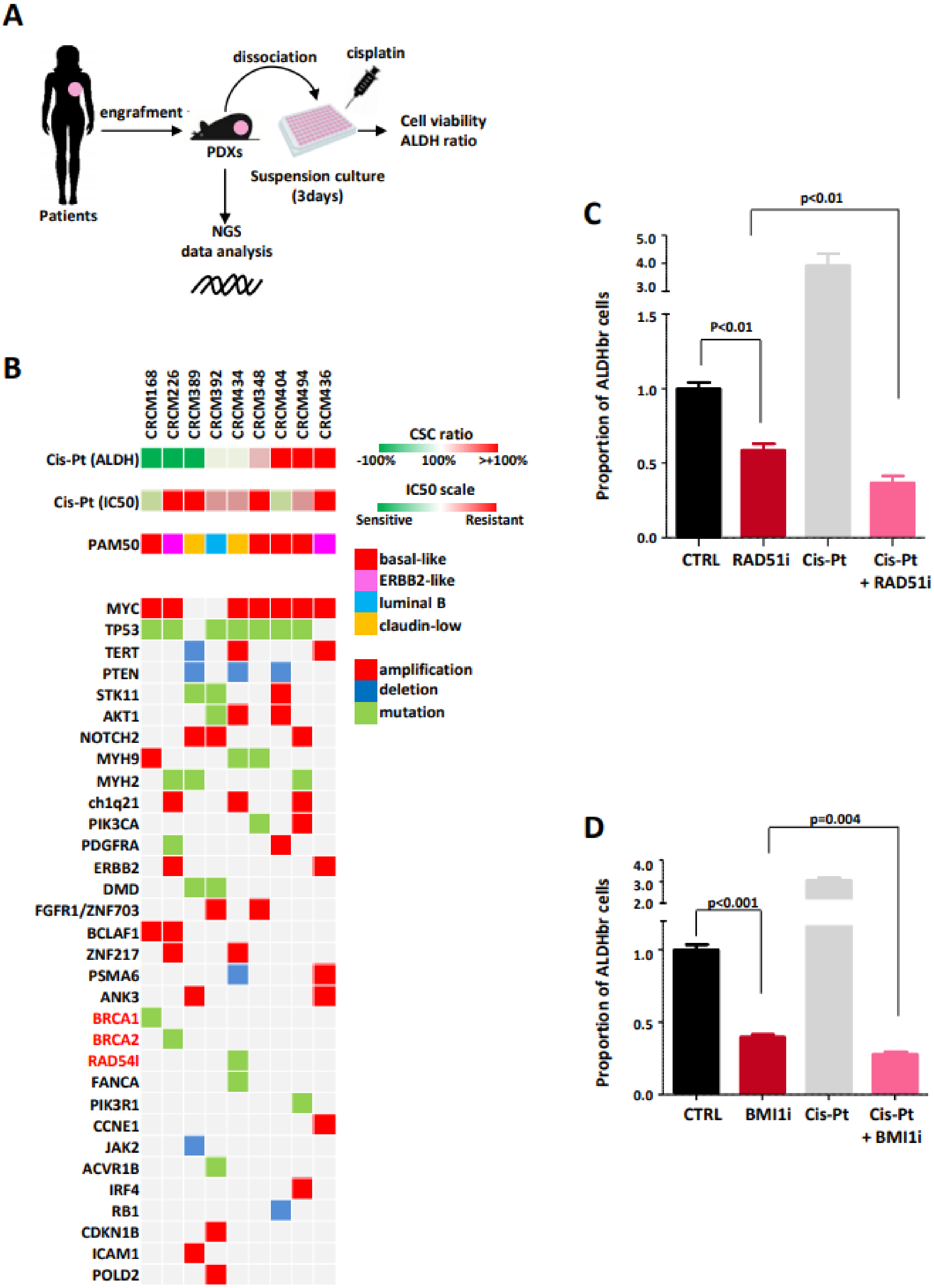
A
Patients
engrafment
PDXs
dissociation
cisplatin
Suspension culture
(3days)
Cell viability
ALDH ratio
NGS
data analysis
B
CRCM168
CRCM226
CRCM389
CRCM392
CRCM434
CRCM348
CRCM404
CRCM494
CRCM436
Cis-Pt (ALDH)
Cis-Pt (IC50)
PAM50
CSC ratio
-100%
100%
>+100%
IC50 scale
Sensitive
Resistant
basal-like
ERBB2-like
luminal B
claudin-low
amplification
deletion
mutation
MYC
TP53
TERT
PTEN
STK11
AKT1
NOTCH2
MYH9
MYH2
ch1q21
PIK3CA
PDGFRA
ERBB2
DMD
FGFR1/ZNF703
BCLAF1
ZNF217
PSMA6
ANK3
BRCA1
BRCA2
RAD54l
FANCA
PIK3R1
CCNE1
JAK2
ACVR1B
IRF4
RB1
CDKN1B
ICAM1
POLD2
C
p<0.01
P<0.01
Proportion of ALDHbr cells
5.0
4.0
3.0
1.5
1.0
0.5
0.0
CTRL
RAD51i
Cis-Pt
Cis-Pt
+ RAD51i
D
p=0.004
p<0.001
Proportion of ALDHbr cells
4.0
3.0
2.0
1.0
0.5
0.0
CTRL
BMI1i
Cis-Pt
Cis-Pt
+ BMI1i

Supplementary Figure 5. Effect of cisplatin treatment alone or in combination with RAD51i/BMI1i on ALDH^{br} bCSC from SUM159 and PDXs. A. Schematic representation of the short-term culture assay design to determine PDX cisplatin treatment response. **B**. The distribution of molecular alterations (green, mutation; red, amplification; blue, deletion) was shown by decreasing frequency across PDX samples. Molecular subtypes calculated with PAM50 algorithm are represented with a color code. Cisplatin response of the tumor bulk (Cis-Pt (IC50)) is color-coded from sensitive PDX in green to resistant in red. Effect of cisplatin treatment on the ALDH^{br} bCSC proportion (Cis-Pt (ALDH)) is color-coded from a 2-fold decrease of the ALDH^{br} bCSC population in the treated condition compared to the untreated (green) to a 2-fold increase (red). **C.** Bar plot representing the proportion of ALDH^{br} cell in SUM159 treated with RAD51i and cisplatin alone or in combination compared to the untreated condition (CTRL). **D.** Bar plot representing the proportion of ALDH^{br} cell in SUM159 treated with BMI1i and cisplatin alone or in combination compared to the untreated condition (CTRL). Statistical test used is Student's t-test. Data represent mean ± SD.

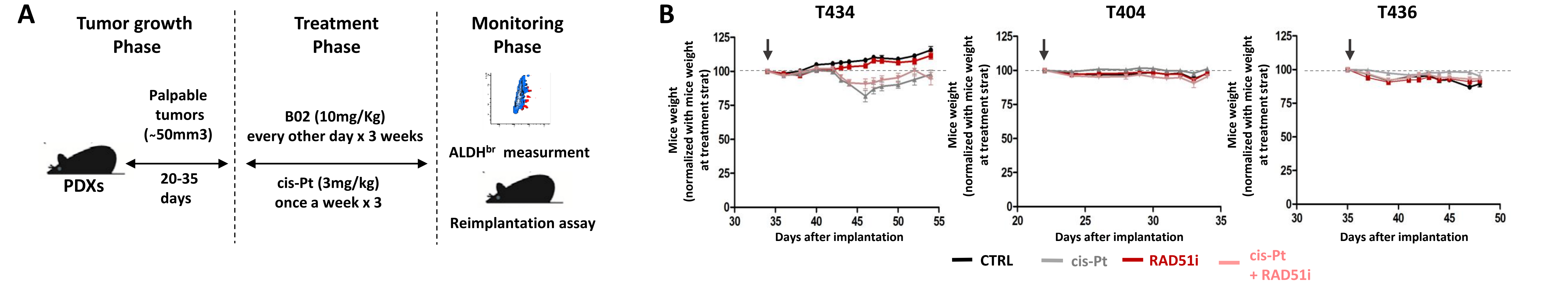

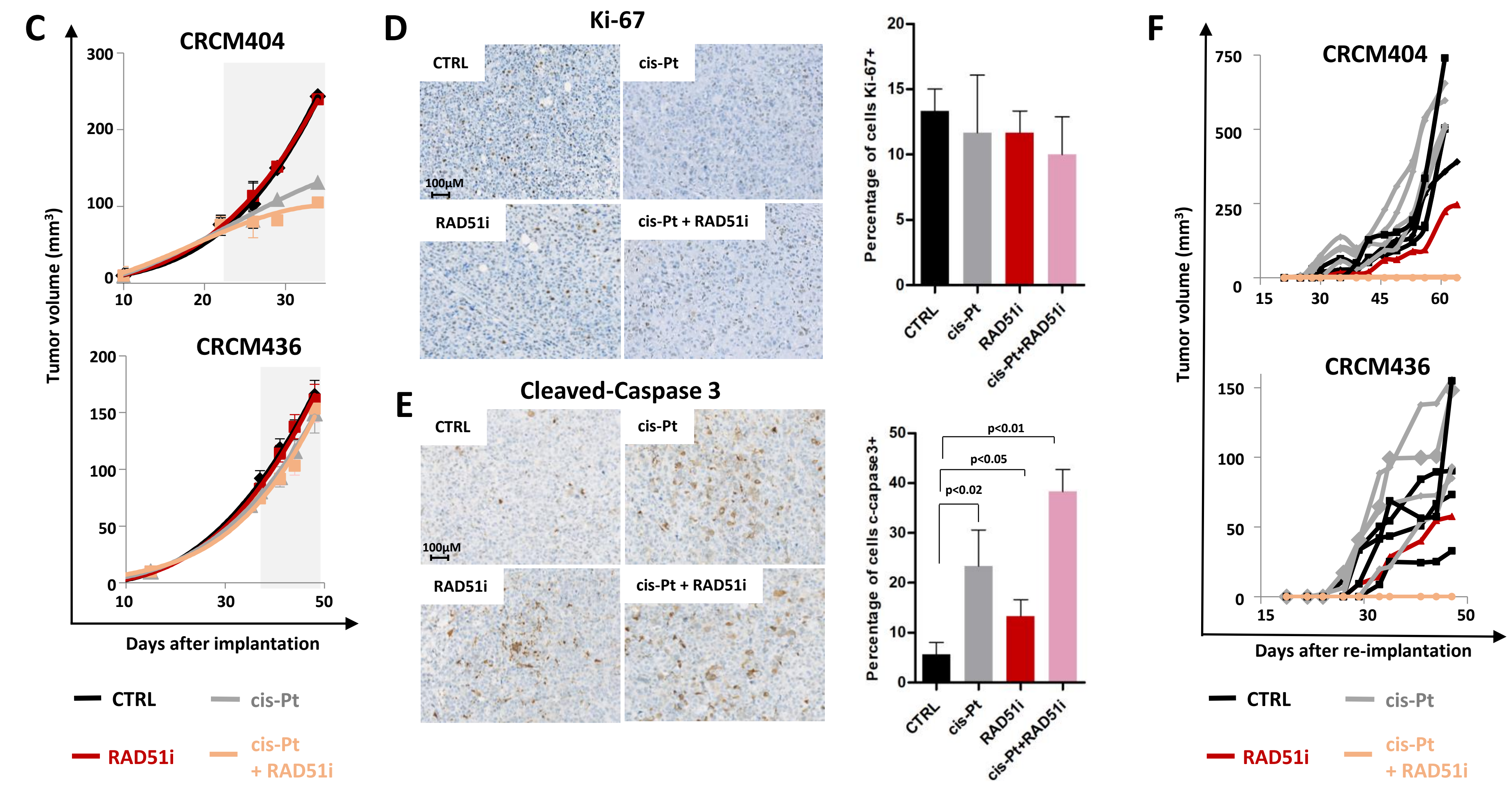

G

	Limiting Dilution Assay (number of injected cells)				
	5	50	500	5,000	50,000
CRCM434 CTRL (outgrowth/injected fatpads)	0/1	4/4	3/3	3/3	
CRCM434 RAD51i (outgrowth/injected fatpads)		0/4	1/3	3/3	
CRCM434 cis-Pt (outgrowth/injected fatpads)		0/4	3/3	3/3	
CRCM434 cis-Pt/RAD51i (outgrowth/injected fatpads)		0/4	0/3	0/3	
CRCM404 CTRL (outgrowth/injected fatpads)		0/1	4/4	3/3	3/3
CRCM404 RAD51i (outgrowth/injected fatpads)			1/4	2/3	3/3
CRCM404 cis-Pt (outgrowth/injected fatpads)		0/1	4/4	3/3	3/3
CRCM404 cis-Pt/RAD51i (outgrowth/injected fatpads)			0/4	2/3	3/3
CRCM436 CTRL (outgrowth/injected fatpads)		0/1	4/4	3/3	3/3
CRCM436 RAD51i (outgrowth/injected fatpads)			1/4	2/3	3/3
CRCM436 cis-Pt (outgrowth/injected fatpads)		0/1	4/4	3/3	3/3
CRCM436 cis-Pt/RAD51i (outgrowth/injected fatpads)			0/4	1/3	3/3

Supplementary Figure 6. Preclinial and reimplantation assays evaluating the proportion of residual bCSC following cisplatin/RAD51i treatments. A. Schematic representation of the in vivo experimental design. **B.** Body weight changes in mice over 15 days of treatment (cis-Pt, RAD51i, cis-Pt+RAD51i, vehicle-treated (CTRL)). *Arrow*: treatment start. **C.** Effect of RAD51i and cisplatin treatment alone or in combination on the tumor growth of CRCM404 and CRCM436, compared to the vehicle-treated condition. The gray area corresponds to the treatment period. **D.** Representative Ki-67 immunostaining (brown staining) in different treated tumors (right panel). Counterstaining in blue. Quantification of the proportion of proliferative Ki-67+ cells (left panel). Data represent mean ±SD (n=3). **E.** Representative cleaved-caspase 3 immunostaining (brown staining) in different treated tumors (right panel). Counterstaining is in blue. Quantification of the proportion of apoptotic cleaved-caspase 3+ cells (left panel). Statistical test used is a t-test. Data represent mean ±SD (n=3). **F.** Reimplantation assay (CRCM404 and CRCM436). Three-week treated PDXs were reimplanted, in serial dilutions, into new recipient mice, and tumor growth was monitored. Each curve represents the growth kinetic from one individual injection. Data represent mean ± SD. **G.** Table showing the number of tumor outgrowths generated in NSG mouse fat pads as a function of the amount of injected cells isolated from CRCM404, CRCM434, and CRCM436 PDXs treated with RAD51i and cisplatin alone or in combination, compared to the vehicle-treated tumors (CTRL).
